# Pneumonia Characteristics in an Intensive Care Unit Setting during and after the COVID-19 Pandemic—A Single-Center Prospective Study

**DOI:** 10.3390/jcm13102824

**Published:** 2024-05-10

**Authors:** Jakub Sleziak, Katarzyna Pilarczyk, Michal Matysiak, Wieslawa Duszynska

**Affiliations:** 1 The Students Scientific Association by Department and Clinic of Anaesthesiology and Intensive Therapy, Wroclaw Medical University, L. Pasteura Street 1, 50-367 Wroclaw, Poland; jakub.sleziak@student.umw.edu.pl (J.S.); katarzyna.pilarczyk@student.umw.edu.pl (K.P.); michal.matysiak@student.umw.edu.pl (M.M.); 2 Department and Clinic of Anaesthesiology and Intensive Therapy, Wroclaw Medical University, L. Pasteura Street 1, 50-367 Wroclaw, Poland

**Keywords:** pneumonia, ventilator-associated pneumonia, intensive care unit, COVID-19, hospital-acquired pneumonia, community-acquired pneumonia, mechanical ventilation

## Abstract

**Background:** During and after the COVID-19 pandemic, there was a suspicion of varying rates of respiratory tract infections (RTIs), particularly pneumonia (PN). **Methods**: This research evaluated epidemiological indicators of community-acquired pneumonia (CAP) and hospital-acquired pneumonia (HAP) in the COVID-19 pandemic and post-pandemic period, including pathogens, ventilator-associated pneumonia (VAP), selected risk factors, and PN mortality. **Results**: At 1740 patients, throughout the 22,774 patient-days (Pt-D) and 18,039 ventilation days (Vt-D), there were 681 PN cases (39.14%): CAP 336 (19.31%) and HAP 345 (19.83%). CAP caused by SARS-CoV-2 was diagnosed in 257/336 (76.49%) patients. The clinical manifestations of PNs were CAP with 336/681 (49.34%), VAP with 232/681 (34.07%), and non-ventilator HAP (NV-HAP) with 113/681 cases (16.59%). The incidence rate of CAP/1000 Pt-D has been over 3 times higher in the pandemic period of 2020–2021 (20.25) than in the post-pandemic period of 2022 (5.86), *p* = 0.000. Similarly, higher incidence rates of VAP/1000 Pt-D were found in the pandemic period (*p* = 0.050). For NV-HAP, this difference was not statistically significant (*p* = 0.585). VAP occurred more frequently in the group of patients with PN in the course of COVID-19 compared to patients without COVID-19 (52/234 [22.2%] vs. 180/1506 [11.95%]); (*p* = 0.000). The most common CAP pathogen (during the pandemic) was SARS CoV-2 234/291 (80.4%), followed by MSSA/MRSA 8/291 (2.75%), whereas the most common VAP/NV-HAP pathogen was *Acinetobacter baumannii* XDR/MDR. The highest PN mortality was found in the patients with CAP caused by SARS-CoV-2 159/257 (61.87%). **Conclusions**: Pneumonias were diagnosed in nearly 40% of Intensive Care Unit (ICU) patients. Surveillance of pneumonias during the specific observation period was beneficial in the epidemiological and microbiological analysis of the ICU patients.

## 1. Introduction

Intensive Care Units (ICU) exhibit the highest prevalence of healthcare-associated infections (HAIs) in comparison to other hospital departments, and nosocomial infections play a crucial role in determining ICU patients’ outcomes [1,2]. Respiratory tract infections (RTI) have been repeatedly indicated as the most common infections among ICU patients (53.73–60.1–64.7%) [3,4,5]. Amid the RTIs occurring in the ICU setting, ventilator-associated pneumonia (VAP), also referred to as intubation-associated pneumonia (IAP) or ventilator-associated events (VAE), were the predominant clinical manifestations in pre-pandemic times [6]. This trend was changed during the pandemic due to a significant increase in patients admitted to ICUs with community-acquired pneumonia (CAP) caused by the SARS-CoV-2 virus [7], which became the predominant clinical form of RTI.

According to the most recently published data, the COVID-19 pandemic is associated with a higher risk of Device-associated Healthcare-associated Infections, overcrowding in ICUs, and increasing numbers of ventilated patients [8,9,10]. Critically ill ICU patients infected by SARS-CoV-2 had a high risk of VAP and bloodstream infection caused by multidrug resistance (MDR) bacterial strains [10]. It was emphasized also in several studies that the mean incidence density of pneumonia at ICUs rises significantly with the percentage of intubated patients [2,11]. The patient’s duration of stay in the ICU, as well as the period of reliance on mechanical ventilation, were also identified as factors that influence pneumonia frequency [12,13]. Risk factors contributing to increased mortality in VAP patients include an age >65, diabetes mellitus, septic shock, chronic obstructive pulmonary disease, secondary peritonitis, SOFA score, and the involvement of high-risk pathogens as etiology [14,15]. A significant majority of NV-HAP (70.8%) originate outside ICUs and 18.8% need a subsequent transfer to the ICU [16].

Microbial factors contributing to pneumonia (PN), especially of hospital origin, can vary depending on geographic region, ICU patients’ specific characteristics, durations of both hospital and ICU stay, and even within different hospital settings [2,17]. In the United States ICUs, the most common etiology factors of VAP were identified in the following descending order of prevalence: *Staphylococcus aureus*, *Pseudomonas aeruginosa*, and *Klebsiella pneumoniae* [18]. In European ICUs, the most frequent pathogens causing VAP were the following: *Klebsiella pneumoniae*, *Staphylococcus aureus,* and *Pseudomonas aeruginosa* [2]. However, in Poland, *Acinetobacter baumannii* emerges as the predominant strain associated with VAP (45%) [7,19,20]. Several studies underlined that in COVID-19 patients, VAP was frequently caused by MDR organisms like methicillin-resistant staphylococcus aureus (MRSA) or Gram-negative bacteria producing extended-spectrum B-lactamase (ESBL) similar to patients without COVID-19 [10,21]. COVID-19-infected VAP patients exhibit higher mortality in comparison to VAP patients without COVID-19 infection (38% vs. 21%, *p* = 0.006) [21]. NV-HAP impacts a larger population than VAP; however, it demonstrates a similar mortality rate [16].

The implementation of prophylactic measures and monitoring standards results in a decrease in the incidence of VAP among mechanically ventilated patients [22].

Due to the COVID-19 pandemic and post-pandemic period observations and possible differences in the prevalence of RTI worldwide, there is an ongoing need for systematic research focused on describing the characteristics, epidemiology, and etiology of pneumonias (the most frequently found RTI) of ICU patients. Additionally, efforts to enhance preventive measures for these infections remain crucial. This study aimed to evaluate the prevalence of different kinds of PN and, additionally, certain risk factors, including microbiological factors, mortality rates, and preventive interventions related to VAP.

## 2. Materials and Methods

### 2.1. Design and Data Collection

From 1 January 2020 to 31 December 2022, a prospective observational study was conducted at the Department of Anesthesiology and Intensive Therapy of the Medical University of Wroclaw. This study contained all patients admitted to the ICU throughout the selected period and covered 1740 patients.

RTIs were identified through regular monitoring of infection. Data collection depended on the monthly reports on ICU infections from the Infection Monitoring and Treatment Laboratory within the Department of Anesthesiology and Intensive Therapy, microbiological data from annual microbiological reports, and a hospital database.

The main objective of this research was to estimate the appearance rates of various clinical manifestations of PN among patients in the ICU. This involved assessing the incidence of PN upon admission (CAP) and throughout the ICU stay, classifying by patient type (surgical and medical) and gender, determining the incidence rates of CAP, VAP, and NV-HAP per 1000 patient-days (Pt-D), and estimating the incidence density of VAP per 1000 ventilator days (VD).

Our study included examining microbiological factors related to infections and assessing conformity with the elements of the VAP prevention protocol named “VAP-bundles”.

The epidemiology nurse gathered information regarding Pt-D and VD. Data on adherence to the elements of the preventive package called “VAP-bundle” were collected twice a week from January 2020 to December 2022 by students from the Students Science Club, following adequate training. Patients with COVID-19 were not included in the “VAP-bundles” observation.

### 2.2. Ethical Approval

All the patients’ data, including microbiology results, involved in this study were received as part of routine patient care and infection monitoring. Adherence to patient data confidentiality was maintained throughout data collection and manuscript preparation, and formal written consent and patient statements were deemed unnecessary by the guidelines set by the Bioethics Committee of the Wroclaw Medical University. Approval of this study is under no. KB-576/2016.

### 2.3. Clinical Diagnosis of RTIs

RTIs were identified following the criteria based on the European Centre for Disease Control and Prevention (ECDC) and the European Network for ICU-related Respiratory Infections (ENIRRIs) project [2,23]. Two doctors working at the ICU (one from the departmental infection control team) together with one microbiologist were responsible for pneumonia diagnosis.

CAP was identified in patients not hospitalized earlier if the clinical symptoms of PN commenced before admission to the ICU.

Hospital-acquired pneumonia (HAP) commenced in the ICU if diagnosed >48 h after admission, or in other hospital wards, if diagnosed before or on the day of admission to the ICU. VAP develops in patients who are on mechanical ventilation, exhibiting symptoms at least 2–3 days following the initiation of ventilation and admission to the ICU. It was identified on the basis of changes in the X-ray imaging, characteristic auscultatory findings, and appropriate biochemical and microbiologic tests.

NV-HAP was identified in a patient not undergoing mechanical ventilation, relying on observed chest X-ray changes, the presence of purulent sputum, changes in auscultation, a raised body temperature above 38 °C, a white blood cell count above 12,000/mm^3^, and the results of relevant microbiological tests (including culture of sputum, blood, pleural fluid cultures, and smears from the pharynx or bronchial secretions collected immediately after intubation with a tracheobronchial tube.

### 2.4. Microbiological Diagnostics Methods

VAP was identified through microbiological analysis of mini bronchoalveolar lavage or bronchoalveolar lavage with a bacterial count ≥10^4^ colony forming unit/mL [24]. The initial microbiological assessment of bronchial secretions involved a polymerase chain reaction multitest for 20 respiratory pathogens (FilmARRAY Respiratory Panel, BioFire Diagnostics, Salt Lake City, UT, USA). Verification of infection was conducted through qualitative and quantitative diagnosis using minimal inhibitory concentrations according to recommendations of the European Committee on Antimicrobial Susceptibility Testing [25,26].

Furthermore, appropriate diagnostic tests from blood or urine were used for Cytomegalovirus, Legionella pneumophilia, and Pneumocystis jiroveci pneumonia identification.

### 2.5. Epidemiological Indicators

The incidence rate of VAP (similarly NV-HAP, CAP) was calculated as the number of individual VAP cases divided by Pt-D × 1000. The incidence density of VAP was calculated as the number of VAP cases divided by the number of VD × 1000. The frequency rate of VAP (similarly NV-HAP, CAP) was calculated as the number of individual pneumonias divided by the number of patients admitted to ICU at 1 year × 100. The ventilation utilization ratio (V-UR) was calculated as the number of mechanical VD divided by the total number of Pt-D × 100.

### 2.6. Statistical Analysis

The statistical analyses were performed using The STATISTICA program version 13.1 (StatSoft Inc., Tulsa, OK, USA). Enumeration data were shown as counts and percentages, and quantitative data which were normally distributed were shown as mean + standard deviation (SD), whereas those that were not normally distributed were shown as median ± interquartile range (IQR) or 95% confidence interval (CI).

These data were analyzed for comparison between groups using as appropriate the chi-square test, or Person’s chi-square test with Yates corrections. *p*-value < 0.05 was considered statistically significant.

## 3. Results

### 3.1. Patients’ Characteristics and General Analysis of Pneumonias

Within the 3-year observational period, there have been 1740 hospitalized ICU patients, 641 of those were female (36.8%) and 1099 were male (63.2%). The number of surgical patients equaled 1112 (64%) and internal medicine ones were 628 (36%). Patients’ characteristics from each year were presented separately in Table 1.

Throughout the 22,774 Pt-D of hospitalization and 18,039 VD, there have been 681 PN cases (39.14%), 336 of those classified as CAP (19.31%), and 345 as HAP (19.83%). The latter comprised 113 (6.49%) NV-HAP cases and 232 (13.33%) VAP cases among admitted patients. From 1 January 2020 to 31 December 2021, COVID-19 with symptoms of severe respiratory failure during CAP caused by SARS-CoV-2 was diagnosed for 234 out of 1168 ICU patients (20.03%), whereas in 2022, it was 23/572 (4.02%); *p* = 0.000.

### 3.2. Comparison of the Amounts of Pneumonias and Its Epidemiological Indicators during the Pandemic and Post-Pandemic Periods

The most common clinical manifestation of pneumonia in 2020–2022 in the ICU was CAP with 336/681 (49.34%) cases, followed by VAP with 232/681 (34.07%) cases, and NV-HAP with 113/681 (16.59%) cases. This study found a vast difference between the amount of CAP cases during the pandemic and post-pandemic period with the highest number in 2021 with 173/296 (58.45%) CI 95% (52.6–63.9%), the least in 2022 with 51/165 (30.91%) CI 95% (23.7–37.7%) cases; *p* = 0.0000. It has been observed that within the pandemic years 2020 and 2021, the total number of PN cases (220 and 296, respectively) was much higher than in 2022 (165). The highest number of VAP cases (92/296 (31.08%) CI95% (25.7–36.2%) occurred in 2021, whereas the highest prevalence was found in 2022 (68/165 (41.21%) CI95% (33.5–48.4%); *p* = 0.0302. Similarly, NV-HAP cases have been the most prevalent in 2022 with 46/165 (27.9%) CI 95% (21.0–34.7%), whereas its least prevalence occurred in 2021 with 31/296 (10.5%) CI 95% (7.0–14.0%); *p* = 0.000. The frequencies of PN cases are presented in Table 2.

The incidence rate of CAP/1000 Pt-D throughout 2020–2022 has been over 3 times higher in the pandemic period of 2020–2021 (20.25) than in the post-pandemic 2022 (5.86); *p* = 0.000. Higher incidence rates of VAP/1000 Pt-D were found in the pandemic period in comparison to 2020 (11.66 vs. 7.81, *p* = 0.050). For NV-HAP, such statistically significant differences were not observed (*p* = 0.5865). Table 3 shows incidence rates of pneumonias/1000 Pt-D.

This study also compared the frequency of CAP, NV-HAP, and VAP/100 admissions to the ICU during 2020–2022 and found significantly higher results for CAP/100 admissions in the pandemic period than in the following year 2022; *p* = 0.000. Such a significant increase was not observed for NV-HAP and VAP frequency; *p* = 0.0675, *p* = 0.2122. Incidence rates of pneumonias/100 admissions to the ICU are presented in Table 4.

The median density of VAP/1000 Vt-D in the 3-year observational period was 11.86 (6.1–17.4) (median ± IQR), and, respectively, in 2020: 9.33 (7.93–13.55), in 2021: 13.61 (8.31–17.04), and in 2022: 12.67 (6.1–14.7). The mean density of VAP/1000 VD in the pandemic period (13.4 ± 7.1) was slightly higher than in the post-pandemic period (11.37 ± 5.5); *p* = 0.2854. Table 5 represents the analysis of the occurrence of VAP during 2020–2022. Ventilation use in the timeline of this study was 76.99%.

### 3.3. VAP Analysis in Relation to Selected Risk Factors

During the analyzed period, VAP (*n* = 232) occurred more frequently in the group of patients with pneumonia in the course of COVID-19 compared to patients without COVID-19 (52/234 (22.1%) CI 95% (16.9–27.5%) vs. 180/1506 (11.95%) CI 95% (10.3–13.6%); *p* = 0.000). Furthermore, it was observed that VAP occurred more frequently in males than in females (176/1099 [16.01%] CI 95% [13.8–18.2%] in males vs. 56/641 [8.74%] CI 95% [6.6–10.9%] in females; *p* = 0.000). Additionally, VAP was more prevalent in internal medicine patients compared to surgical patients (151/628 [24.04%] CI 95% [20.7–27.4%] vs. 81/1112 [7.28%] CI 95% [5.8–8.8%]; *p* = 0.000).

**Figure 1:**
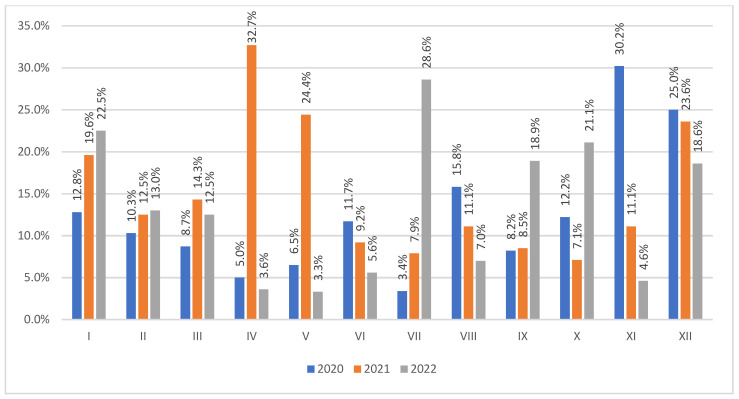
The percent number of VAP patients out of the admitted patients in each month of 2020–2022. VAP—ventilator-associated pneumonia.

Figure 1 shows the percentage of VAP patients out of the patients admitted to the ICU monthly in a given year. The most cases were observed in XI (16/53, 30.2%) in 2020, IV (17/52, 32.7%) in 2021, and VII (10/32, 28.6%) in 2022.

### 3.4. Microbiological Analysis of Pneumonias Pathogens

The most common CAP pathogen in the pandemic period was the virus SARS-CoV-2 with 234/291 (80.4%), followed by MSSA/MRSA with 8/291 (2.7%), *Streptococcus pneumoniae* with 7/291 (2.4%), and, in similar levels, 5/291 (1.7%) for influenza virus A, *Haemophilus influenzae*, and *Klebsiella pneumoniae,* whereas in 2022, it was SARS-CoV-2 with 23/68 (33.8%), *Streptococcus pneumoniae* with 11/68 (16.2%), MSSA/MRSA with 6/68 (8.8%), and both influenza virus A and *Haemophilus influenzae* with 5/68 (7.4%).

The most common VAP pathogen strain throughout the observational period was Acinetobacter baumannii XDR/MDR. It was followed by *Klebsiella pneumoniae* and *Pseudomonas aeruginosa*. Full data are shown in Table 6.

Gram-negative bacteria with metallo-β-lactamases (MBL) resistance mechanism have been associated with 15/200 (7.5%) CI 95% (3.8–11.1%) isolated pathogen strains in the pandemic period and 16/89 (18%) CI 95% (9.9–25.7%) ones in the post-pandemic one (*p* = 0.0084).

The most frequently found pathogens responsible for NV-HAP in the pandemic period were *Acinetobacter baumannii* XDR/MDR 16/97 (16.5%), *Klebsiella pneumoniae* 11/97 (11.3%), and *Pseudomonas aeruginosa* XDR/MDR 7/97 (7.2%), whereas in 2022, it was *Klebsiella pneumoniae* 8/57 (14%), *Acinetobacter baumannii* XDR/MDR and both MRSA/MSSA in 7/57 (12.3%), and both *Pseudomonas aeruginosa* and *Stenotrophomonas maltophila* in 6/57 (10.5%).

### 3.5. Analysis of VAP Preventive Interventions Implementation

This study analyzed the implementation of preventive packages for VAP according to CDC guidelines. The “ventilation ducts devoid of bronchial secretions” has been the most fulfilled criteria with 95% implementation. It was followed by “prevention of stress ulcers” with 89% implementation. The lowest implementation percentage has been noted for “regular subglottic suction” at 31% [Table 7].

### 3.6. Mortality Rates Assessment for Selected Kinds of Pneumonia

This study showed that 109/232 (46.98%) patients with VAP and 34/113 (30.09%) with NV-HAP died. Very high mortality was found in the patients with CAP caused by SARS-CoV-2 159/257 (61.87%).

## 4. Discussion

### 4.1. General Analysis of Pneumonias

The study results indicate that PN posed a significant epidemiological challenge, as it was diagnosed in almost 40% of ICU patients. In this study, due to the inclusion of the SARS-CoV-2 pandemic period, the most commonly found clinical form of PN was CAP, which attributed to almost half of the observed PN cases (49.34%). As it was a global problem, many articles in this area underlined a very high frequency of CAP (caused by SARS-CoV-2) found at admission to ICUs and other hospital departments [21,27,28]. Along with NV-HAP observed in patients at the moment of admission to the unit, it accounted for the majority (62.6%) of PN cases at the ICU, which corresponds with the findings of PN epidemiology at the same unit in pre-pandemic times (CAP and NV-HAP at admission to ICU), constituting to 55% (76/136) PN episodes [6]. The distinction in these findings lies in the fact that, in our study, CAP was a more frequent form of PN at the moment of admission to the department in comparison to the previous research (78.9% vs. 44.7%) (the *p*-value < 0.00001), which reflects the unique characteristics of the study period [6].

Our findings align with those of the multi-center European (EU) VAP/CAP study, where CAP was diagnosed less frequently, accounting for 10.75% (262/2436), compared to all types of HAP combined, which had a higher frequency at 33.95% (827/2436) [15]. The same relationship is consistent with the previous research in our department in 2018: CAP 5.88% (34/578) and HAP 17.65% (102/578) hospitalized patients [6].

According to our findings, NV-HAP occurred less frequently during the ICU stays (20.4% [23/113]) than at admission to the ICU, which is consistent with the previous observations (19.2% [10/52] vs. 80.8% [42/52]) [6], as well as with findings of a recent ENIRRI’s multicentric and observational cohort study (28.5% [98/344] vs. 71.5% [246/344]) [29].

The consistently high percentage of NV-HAP diagnosed upon admission to the ICU from other hospital departments necessitates continuous preventive measures at the hospital level. The ENIRRIs’ research indicated VAP as the most common form of HAP, which is consistent with our findings [29].

### 4.2. Analysis of Pneumonia Diagnosed during ICU Stay in the Pandemic and Post-Pandemic Period

In the observed period, PN developed in 14.7% (232/1740) of patients during hospitalization in the ICU, which is significantly more than the 3.9% (4706/120,446) observed in 2019 in mainly Western European countries by the ECDC (*p*-value < 0.00001) [2].

The incidence rate of ICU HAP (number of VAP + NV-HAP), when measured per 1000 Pt-D, was 11.2, which slightly exceeds the incidence rate of 10.3/1000 Pt-D established in the same center in 2018 [6].

This rate is also more than twice as high as the incidence of 4.4 ICU pneumonia episodes per 1000 Pt-D (IQR:0.0–6.3) listed in the ECDC 2019 registry [2], as well as in the corresponding 2018 report (3.7 pneumonia episodes per 1000 Pt-D (IQR:0.7–4.6) [30]. However, no such report has so far been released for the time interval equivalent to ours. 

The mean incidence density of VAP/1000 Vt-D during the observed 3-year period in this study (12.49 ± 6.6; 11.86 [6.1–17.4] [mean ± SD, median ± IQR]) exceeds the average value from the ECDC 2019 register (7.8 intubation-associated pneumonia episodes per 1000 intubation days); however, the spread of this indicator varied between 2.5 in the UK–Scotland, and 14.4 in Belgium [2]. Moreover, our findings exceed the incidence density of 10.8, IQR (8.5–12.32) VAP/1000 VD found in 2018 in our department [6]. Throughout the years 2015–2017 VAP incidence density [(median (IQR)]/1000 VD was 13.66 (12.01–13.77) in our unit [20] and collectively in the period from 1 January 2011 to 31 June 2019 11.15 ± 2.5 [mean ± SD] [31].

A multinational prospective cohort study conducted by the INICC over 24 years (between 1 July 1998 and 12 February 2022) determined the incidence density of VAP in Poland (95% CI) of 17.27 (17.20–17.33)/1000 VD and for high-income country’s university hospitals’ VAP rate (95% CI) of 13.22 (13.15–13.28) [13]. V-UR for this INICC study was 66.43% for Poland and 70.93% for high-income university hospitals [13], which is less than the V-UR observed in our study (76.99%). The EU VAP/CAP study found a VAP density of 18.3 episodes of VAP per 1000 VD [15].

In the NHSN register, the rate of VAE/1000 VD in the years 2020–2022 was 9.286 [32]. These rates for particular years in this register are present as follows: 2020: 8.988; 2021: 10.078; and 2022: 8.589 [32]. Each year’s rate is less than our findings for the corresponding years (mean ± SD, median ± IQR): 2020: 12.60 ± 8.5; 9.33 (7.93–13.55); 2021: 13.50 ± 5.7; 13.61 (8.31–17.04); 2022: 11.37 ± 5.5, 12.67 (6.1–14.7). It is worth mentioning that during the post-pandemic period, a decrease in the incidence density of VAP was observed, along with a decline in the frequencies of CAP and NV-HAP. We suspect a decreasing tendency in VAP frequency at our ICU in the next year with an increasing role of MDR Gram-negative bacteria. The next study in this area with an assessment of different VAP risk factors will be provided. Nonetheless, the high frequency/density of VAP occurrence necessitates an improvement in the implementation of established preventive measures.

Moreover, in the EU VAP/CAP study, the established density of VAP of 18.3 was significantly (54%) higher than the one found in our study (11.86).

Considering the density of VAP/1000 VD at our ICU, an increasing tendency was observed especially during the pandemic period. This observation is in accordance with other published data in which it was highlighted that in COVID patients the VAP density increased, and, in Wickly et al.’s study, it even reached 46.5/1000 VD [33]. In a Polish study (2020–2021), VAP incidence rate density in COVID-19 patients was also higher than in non-COVID-19 patients and reached 14.1/1000 Pt-D [7], whereas in an Italian study, it was 26.03/1000 Pt-D [10]. 

### 4.3. Pneumonias Mortality Analysis 

According to INICC reports, the crude mortality rate among ICU patients without HAI is 17.12% (95% CI, 16.93–17.32), while for those with VAP, it increases to 42.32% (95% CI, 40.61–44.09) [34] and is lower than the 47% found in our study. The high mortality in our study resulted from the fact that in this specific period, VAP was diagnosed mainly in patients with COVID-19. In our study, the mortality in NV-HAP patients equals around 1/3. A prospective, multicentric, and observational cohort study at 28 selected ICUs in 13 countries across Europe and Latin America found that among patients with nosocomial lower respiratory tract infections in the ICU, those with HAP requiring following invasive ventilation experience the highest mortality rate [29]. The mortality rate of CAP caused by COVID-19 found in our study (62%) slightly exceeds the mortality of 60% established in a large meta-analysis that pooled still-hospitalized ICU COVID-19-infected patients with non-survivers, which Chang et. al called “the worst-case scenario” and is more than twice greater than the 28.3% found in the same study by pooling patients only with the recorded outcome, which authors called “the best case scenario” [35]. Nevertheless, the mortality rates vary from 25.7% [36] to 78% [37], depending on the facility. This mortality rate established in our research is similar to the rate of 64% found by Wałaszek et. al in the same period in another Polish hospital [7].

### 4.4. VAP Analysis in Relation to Selected Risk Factors

During the analyzed period, VAP occurred more frequently in the group of patients with pneumonia caused by COVID-19 than in patients without COVID-19 (52/234, 22.1% vs. 180/1506, 11.95%; *p* = 0.000). This corresponds to the findings of other research where the incidence density of VAP during the COVID-19 period exceeded that of the pre-COVID-19 period (19.3 vs. 27.8 per 1000 VD); however, no statistical significance was found [38]. 

In a study conducted in 2020 and 2021 at an ICU department of a hospital in Poland, VAP occurred with an incidence density rate of 6.3/1000 Pt-D: 14.1/1000 Pt-D for COVID-19 patients versus 3.6/1000 Pt-D for non-COVID-19 patients. The Odds Ratio (OR) was 2.297, indicating a significantly higher risk of VAP in COVID-19 patients (*p*  <  0.01), which agrees with our findings [7]. According to our study, VAP was most frequently found in male internal patients, which seems to be concomitant with the COVID period in which CAP was found more frequently in males [39,40], as well as with the fact that community-acquired setting of COVID infection does not imply surgical profile of patients.

### 4.5. Microbiological Analysis of CAP, VAP, NV-HAP Pathogens

Our microbiological findings of VAP pathogens differ from data collected from various locations. ENIRRIs research revealed the following pathogens as the most frequent etiologic factors isolated from VAP patients (*n*  =  419): *P. aeruginosa* (18.2%), *Staphylococcus aureus* (13.9%), *Acinetobacter baumannii* (11.9%), and *Klebsiella* spp. (10.3%) [29]. CDC NHSN HAI pathogens report in USA’s Acute Care Hospitals 2018–2021 found *Staphylococcus aureus* as the most commonly found pathogen in possible ventilator-associated pneumonia (PVAP) patients (29.6%), followed by *Pseudomonas aeruginosa* (13.4%) and *Klebsiella* spp. (12.1%) [41]. The *Acinetobacter* spp. was the tenth most frequent etiologic factor for PVAP; however, it had the highest resistance percentage (36.1% of Carbapenem non-susceptibility) [41]. During the COVID-19 pandemic, the most common pathogens causing VAP in our study were identified as *Acinetobacter baumannii* XDR/MDR, with a prevalence ranging from 40.78% to 54.17%, followed by *Klebsiella pneumoniae* (11.1% to 21.29%, including *Klebsiella pneumoniae* MDR/XDR/MBL at 9.7%). Moreover, in another Polish ICU department in 2020 and 2021, the predominant microorganism in VAP cases was also *Acinetobacter baumannii* with an incidence rate of 8.5%, which was notably higher in COVID-19 patients [7].

In the post-pandemic period, this trend was not changed and again the most common pathogens causing VAP were *Acinetobacter baumannii* XDR, accounting for 33.7%, followed by *Klebsiella pneumoniae* at 28.1% and *Pseudomonas aeruginosa* at 10.15%. 

When comparing to findings of the previous study in our facility, the VAP etiology remains almost uniform, again with the most common pathogen for VAP being *Acinetobacter baumannii* MDR (45%), *Klebsiella pneumoniae* ESBL+ (14%) with a difference in that MSSA incidence of 9% was slightly higher than *Pseudomonas aeruginosa* (8%) [6]. It is worth saying that *Pseudomonas aeruginosa* was more frequently responsible for VAP than any other HAIs in our department [31]. Other analyses confirm that *Acinetobacter baumannii* remains the most common etiology of VAP in our unit [20,42].

A significantly higher frequency of isolation of multidrug-resistant pathogens was observed among VAP patients compared to other nosocomial lower respiratory tract infections [29]. The high percentage of multidrug-resistant pathogens in the pathogenesis of respiratory infections requires constant preventive measures at the hospital and ward levels.

### 4.6. Analysis of VAP Preventive Interventions Implementation

The final aspect of this study involved assessing components utilized in the prevention of VAP. The prevention bundle encompasses practices such as employing subglottic suction, utilizing chlorhexidine for oral hygiene, elevating the bed head by 30–50%, maintaining endotracheal/tracheostomy tube balloon pressure above 20 cm H_2_O, ensuring ventilation duct hygiene, adopting a sedation protocol with daily awakening, employing anticoagulant prophylaxis, and administering stress ulcer prophylaxis. Recent systematic reviews confirm that the implementation of ventilator care bundles decreases VAP incidence [22,43] and reduces treatment costs [44]. The introduction of evidence-based bundle measures in Spanish ICUs led to a noteworthy reduction of over 50% in the incidence of VAP. The adjusted incidence density rate decreased from 9.83 (95% CI, 8.42–11.48) per 1000 VD during the baseline period to 4.34 (95% CI, 3.22–5.84) after 19–21 months of participation [45]. The infrequent implementation of subglottic suction in our study was due to the periodic unavailability of intubation tubes equipped with subglottic suction capability. In this study, VAP compliance ranges were 95%, whereas they were 96.2–76.8% in 2015–2017 in our department [20].

### 4.7. Limitations of the Study

The study has several limitations. Being a single-center study, the frequency of pneumonia and the microbiological profile of the ward may differ from other healthcare centers. Furthermore, a comprehensive comparison of various elements of this study, such as the incidence of VAP in women, men, and general surgery patients, was not possible due to the absence of published ICU studies in this specific area. Moreover, the impact of adherence to bundle elements on the frequency of VAP was not analyzed, as this aspect was not included in the study’s assumptions.

It is important to note a potential limitation in our study as we compared our data with European and USA data on pneumonia monitoring. The studies we referenced utilized slightly different diagnostic methods for VAP and VAE and variations in diagnostic approaches across studies may impact the comparability of the findings.

We recommend further the multi-center approach for investigating characteristics, risk factors, and preventative measures against pneumonia occurring in the ICU setting. Comparing data from multiple departments can provide significant insight into this important issue. Moreover, microbiological profiles of etiological factors of such infections should be monitored and reported since the high frequency of MDR organisms poses a serious threat to the future efficacy of available antibiotics.

## 5. Conclusions

Pneumonias were diagnosed in nearly 40% of patients hospitalized at the ICU. CAP caused by SARS-CoV-2 was the most the most frequent. The density of VAP/1000 VD was higher in the pandemic period. VAP was the most prevalent in males and internal medicine patients, as well as in COVID patients. The most common VAP and NV-HAP pathogen was *Acinetobacter baumannii* XDR/MDR, followed by *Klebsiella pneumoniae*.

Surveillance of pneumonias during the specific observation period was beneficial in the epidemiological and microbiological analysis of the ICU patients. Compliance with particular CDC VAP preventive criteria needs improvement.

## Figures and Tables

**Table 1:** Patients’ characteristics.

Year	2020	2021	2022
Total number of hospitalized patients; *n*	570	598	572
Women; *n* (%)	210 (36.8)	226 (37.8)	205 (35.8)
Men; *n* (%)	360 (63.2)	372 (62.2)	367 (64.2)
Surgical patients; *n* (%)	339 (59.5)	342 (57.2)	431 (75.3)
Internal medicine patients; *n* (%)	231 (40.5)	256 (42.8)	141 (24.7)
Total number of patient-days; (*n*)	6517	7553	8704

**Table 2:** Frequency of hospital-acquired and community-acquired pneumonia cases during 2020–2022.

Year	2020	2021	2022
CAP; *n* (%)	112 (50.9)	173 (58.4)	51 (30.9)
NV-HAP; *n* (%)	36 (16.3)	31 (10.5)	46 (29.9)
VAP; *n* (%)	72 (32.7)	92 (31.08)	68 (41.2)
Total, *n*	220	296	165

CAP—community-acquired pneumonia; NV-HAP—non-ventilator hospital-acquired pneumonia; VAP—ventilator-associated pneumonia.

**Table 3:** Incidence rates of CAP, NV-HAP, VAP/1000 patient-days during 2020–2022.

Year	Pandemic Period (2020–21)	Post-Pandemic Period (2022)
CAP	20.25	5.86
NV-HAP	5.6	3.79
VAP	11.66	7.81
Total number of patient-days; (*n*)	14,070	8704

CAP—community-acquired pneumonia; NV-HAP—non-ventilator hospital-acquired pneumonia; VAP—ventilator-associated pneumonia.

**Table 4:** Incidence frequency of CAP, NV-HAP, VAP/100 admissions to ICU during 2020–2022.

Year	Pandemic Period (2020–21)	Post-Pandemic Period (2022)
CAP	24.4	8.9
NV-HAP	6.85	5.77
VAP	14.04	11.9
Total No. of patients	1168	572

CAP—community-acquired pneumonia; NV-HAP—non-ventilator hospital-acquired pneumonia; VAP—ventilator-associated pneumonia.

**Table 5:** Analysis of the occurrence of VAP during 2020–2022. Data are presented as mean ± standard deviation, %, or number.

Year	Pandemic Period (2020–2021)	Post-Pandemic Period (2022)
Density of VAP/1000 ventilation days	12.60 ± 8.5 (2020) 13.50 ± 5.7 (2021)	11.37 ± 5.5
Days of ventilation	5615 (2020) 6548 (2021)	5876
Number of patients with VAP	72 (2020) 92 (2021)	68
Ventilation use %	86.39 ± 5	67.6 ± 9

VAP—ventilator-associated pneumonia.

**Table 6:** Pathogens of ventilator-associated pneumonia. Data are presented as a number of pathogens and % from the total number of strains responsible for VAP during the pandemic period (*n* = 200) and post-pandemic period (*n* = 89).

Pandemic Period (2020–2021)	Post-Pandemic Period (2022)
*Acinetobacter baumannii* XDR/MDR 79; 39.5%	*Acinetobacter baumannii* XDR/MDR 30; 33.7%,
*Klebsiella pneumoniae* 29; 14.5%; including MBL 5%	*Klebsiella pneumoniae* 25; 28.1%; including MBL 15.8%
*Pseudomonas aeruginosa* 15; 7.5%; including MBL 2.5%	*Pseudomonas aeruginosa* 9; 10.1%; including MBL 2.3%
*MRSA* 13; 2.5%	*MRSA* 5; 5.6%
*Stenotrophomonas maltophila* 13; 2.5%	*Aspergilluss* spp. 3; 3.4%
*Candida* spp. 8; 4%	*Candida* spp. 3; 3.4%
*Enterobacter cloacae* 8; 4%	*Enterococcus faecalis* 2; 2.2%
*Escherichia coli* 6; 3%	*Stenotrophomonas maltophila* 2; 2.2%
*Proteus mirabilis* 5; 2.5%	*Citrobacter freundii* 2; 2.2%
Others 24; 12%	Others 8; 9%

XDR—extensively drug-resistant; MDR—multiple drug resistant; MBL—Metallo-β-lactamase; MRSA—Methicillin-resistant Staphylococcus Aureus.

**Table 7:** Analysis of the implementation of preventive packages for VAP according to CDC guidelines. The results are presented as a percentage of completion from the total number (*n* = 1130) of observations.

Type of Observation	Percentage of Implementation of Recommendations (%)
Ventilation ducts devoid of bronchial secretions	95
Prevention of stress ulcers	89
Deep vein thrombosis prophylaxis	84
Endotracheal cuff pressure between 20 and 30 cm H_2_O	81
45° elevation of head of the bed	79
Oral rinse with disinfectant	70
Performed assessments of readiness to wean	37
Regular subglottic suction	31

## Data Availability

The data collected, analyzed, and presented during this study are available on request from the corresponding author, who can be accessed via e-mail at wieslawa.duszynska@umw.edu.pl.

## Abbreviations

CAP—Community-acquired Pneumonia; CI—coincidence interval; ENIRRIs—European Network for ICU-related Respiratory Infections; EU—European; ESBL—extended-spectrum beta-lactamases; HAI—healthcare-associated infections; HAP—Hospital-acquired pneumonia; ICU—Intensive Care Unit; INICC—International Nosocomial Infection Control Consortium; IQR—interquartile range; LOS—length of stay; MDR—multidrug resistant; MIC—Minimal Inhibitory Concentrations; MRSA—methicillin-resistant Staphylococcus aureus; MSSA—methicillin-sensitive Staphylococcus aureus; NHSN—National Healthcare Safety Network; NV-HAP—Non-ventilator Hospital-acquired Pneumonia; PN—pneumonia; Pt-D—patient-days; PVAP—possible Ventilator-associated Pneumonia; RTI—Respiratory Tract Infection; SD—standard deviation; VAE—Ventilator-associated Event; VAP—Ventilator-associated Pneumonia; VD—ventilation days; V-UR—ventilation utilization ratio; XDR—extensively drug-resistant.
